# Severe leukocytoclastic vasculitis secondary to the use of a naproxen and requiring amputation: a case report

**DOI:** 10.1186/1752-1947-4-204

**Published:** 2010-07-01

**Authors:** Keri Brown, Jeanine Martin, Susan Zito

**Affiliations:** 1 HCA Largo Medical Center, Indian Rocks Road, Largo, Florida, 33774, USA

## Abstract

**Introduction:**

Leukocytoclastic vasculitis (also known as hypersensitivity vasculitis and cutaneous necrotizing vasculitis) can present with various manifestations, which often delays the diagnosis and treatment. In order to show the importance of the early recognition of leukocytoclastic vasculitis, we present a case which occurred secondary to the use of a common pharmaceutical, naproxen. We were unable to find a case of leukocytoclastic vasculitis secondary to naproxen in the literature.

**Case presentation:**

We present the case of a 33-year-old African American woman with below the knee and bilateral digital gangrene from hypersensitivity vasculitis secondary to the non-steroidal anti-inflammatory medication naproxen.

**Conclusion:**

This is an original case report focusing on the rheumatologic management of leukocytoclastic vasculitis. However, other specialties, such as internal medicine, dermatology, infectious disease, general surgery and pathology, can gain valuable information by reviewing this case report. Reporting a case of leukocytoclastic vasculitis secondary to treatment with naproxen will advance our understanding of this disease etiology by adding yet another non-steroidal anti-inflammatory drug to the list of potential causes of leukocytoclastic vasculitis.

## Introduction

The term leukocytoclastic vasculitis (LCV) has been used interchangeably with other descriptions of small-vessel vasculitides, including drug-induced vasculitis, allergic vasculitis, hypersensitivity angiitis, and hypersensitivity vasculitis (HSV) [1]. Leucocytoclastic angiitis is an isolated condition without systemic vasculitis or glomerulonephritis [2,3]. The American College of Rheumatology (ACR) has developed criteria for the classification of hypersensitivy vasculitis [4,1]. When used diagnostically the positive predictive value of these criteria is only ~30% [1]. In addition, other limitations include the lack of differentiation of hypersensitivity vasculitis from Henoch-Schönlein purpura (HSP) [1]. Michel *et al*. proposed a different classification methodology using the same database of patients with vasculitis as that used for the ACR criteria to differentiate HSV from HSP [1]. Using this classification, the presence of three or more of these criteria (see Additional File 1), has a sensitivity of 71% and a specificity of 84% for the diagnosis of HSV [1].

The inflammation of small blood vessels, most commonly postcapillary venules, is the cardinal histologic feature of LCV [1]. Other characteristic features include fibrinoid necrosis of the vessel walls, leukocytoclasis, and hemorrhage [1,3]. The inflammatory infiltrate is typically neutrophilic; other studies have shown a predominance of mononuclear cells and eosinophils distributed in all vessel layers [1]. Direct immunofluorescence has been detected in LCV, in which early stages of vascular injury have fibrinogen, C3, and immunoglobulin M deposits have been detected in the vessel wall [1]. In fully manifest lesions, albumin, fibrinogen, and IgG deposits are present; lesions at later stages have fibrinogen and C3 deposited in vessel walls [1,5]. The severity of the lesion may correlate with disease course [1]. The presence of palpable purpura correlates with a greater depth of inflammatory infiltrate and self-limited disease [1,6]. However, vessel wall inflammation was not associated with the presence or absence of systemic vasculitis [1].

The skin is the most commonly involved organ in LCV, predominantly in the lower extremities [1,7,8]. Up to one-third of patients have trunk and upper extremity involvement, typically sparing the palmar, plantar, and mucosal surfaces [1,8]. The most common skin manifestation and the most sensitive finding of HSV is palpable purpura [1,7,8]. Other skin manifestations include maculopapular rash, bullae, papules, plaques, nodules, ulcers, and livedo reticularis [1,8]. Other manifestations of LCV include joint involvement [1,8]. Some patients may have arthralgias or arthritis as the presenting symptom, usually oligoarthritis of the knees or ankles [1,8]. Systemic manifestations of LCV are much less common than its dermatologic features. These include fever, microscopic hematuria, elevated creatinine, pericarditis and pleuritis [1,9].

Laboratory data in the evaluation of LCV is useful in excluding other vasculitides, but there is no specific laboratory test for LCV [1]. Elevation of erythrocyte sedimentation rate (ESR) is often seen in HSV [1,8]. A small amount of patients will have anemia, while complement levels are typically normal in LCV [1,8]. Most patients with LCV will have negative antinuclear antibodies, rheumatoid factors, antibodies to human immunodeficiency virus (HIV), antinuclear cytoplasmic antibodies, and cryoglobulins [1,7,8].

In the pathogenesis of HSV, circulating immune complexes with soluble antigens, either intrinsic or extrinsic, are deposited into vessel walls and activate the classic and alternative complement pathways [1]. HSV is thought to occur through this mechanism with a drug acting as a hapten and stimulating an immune response [1,10].

Numerous series and case reports have reported drugs as the precipitating agent in LCV [1,8,11]. In patients with biopsy proven LCV, 24% had drug exposure within a week of presentation [1,7]. There are many drugs that have been implicated in the development of LCV including antibiotics, non-steroidal anti-inflammatory drugs (NSAIDs), methotrexate, azathioprine, etanercept, cyclosporine, allopurinol, sulfasalazine, gold salts, antithyroid agents, anticonvulsants, antiarrhythmics, and diuretics [1]. Most cases of LCV are self-limited, lasting several weeks to months [1,11]. One study showed that 90% of patients with HSV had resolution of symptoms in less than one year [1,8]. Also, most patients with LCV have complete recovery without sequelae [1,7]. Most patients with an acute episode of HSV do not require treatment [1]. Patients with an identifiable precipitating etiology have outcomes similar to that of patients without a known trigger [1,8]. Patients with palpable purpura have a better disease outcome and course, as compared to patients with vasculitic skin ulcers [1,6].

The treatment of HSV includes discontinuing any causative medication [1]. If the identification of a single drug is not possible then there should be an attempt to withdraw as many suspected etiologies as possible [1]. Agents involved in the treatment of LCV are NSAIDs, colchicine, dapsone, corticosteroids, cyclophosphamide, azathioprine, plasma exchange, and intravenous immune globulin [1].

## Case presentation

A 33-year-old African American woman presented to the emergency medical services of our hospital complaining of a chest pain that began suddenly that morning while she was resting in bed. She described the chest pain as sharp and non-radiating in the peristernal area. Deep breaths and movement exacerbated her chest pain. Nitroglycerin given sublingually partially relieved the pain. On initial presentation she also complained of right foot pain progressively worsening over the past five days. She also admitted to bilateral hand pain worsening over the past three days. The right foot pain prompted an emergency room visit one week prior to this admission for which she was treated with naproxen [Naprosyn] and propoxyphene and acetaminophen [Darvocet], which gave her minimal relief of the pain in her right foot.

Her medical history is significant only for normal childhood illnesses. Her surgical history only includes a cesarean section. Socially she admitted to drinking two bottles of beer on a daily basis but denied tobacco or drug use. Her father died at the age of 46 secondary to a myocardial infarction, and her mother is alive with hypercholesterolemia, diabetes, hypertension and arthritis. She also has a brother with diabetes. She denies food, drug or environmental allergies. Her home medication includes naproxen and propoxyphene and acetaminophen, which were prescribed to her one week prior to this admission.

**Figure 1:**
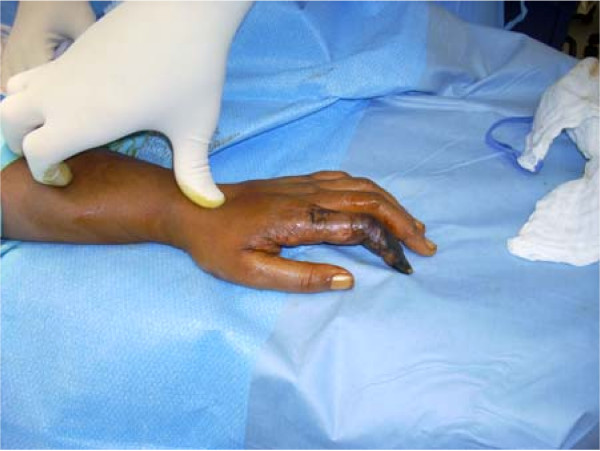
**Digital Gangrene of the left index finger**. Digital gangrene on the left index finger prior to amputation on day 30 of admission. Our patient signed consent for photographs to be obtained.

**Figure 2:**
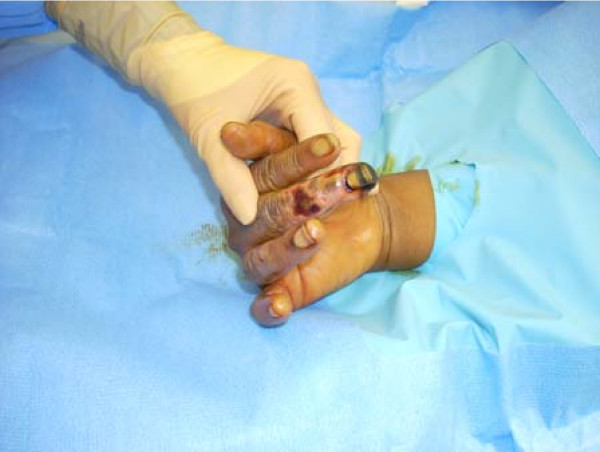
**Digital gangrene of the right middle finger**. Digital gangrene on right middle finger prior to amputation on day 30 of admission. Our patient signed consent for photographs to be obtained.

On initial physical examination she was noted as having violaceous purpuric patches and macules that were non-palpable, non-blanchable lesions distributed bilaterally over her hands and right foot. These lesions were consistent with vasculitis or arterial insufficiency. Her toes and midfoot were completely involved in a stocking pattern with sparing at the heel. Her ankle to middle leg exhibited coalescent ecchymoses and purpura in an incompletely circumscribed distribution around the limb. The upper margins demonstrated scattered petechiae extending to just below her knee. Her popliteal, posterior tibial and dorsalis pedis pulses were palpable. Her left second finger also had violaceous, coalescing, purpuric macules and petechiae extending into the dorsal space between her thumb and second finger. All of her involved extremities were extremely painful to touch or motion with non-pitting edema of moderate degree. Photographs of her involved digits were obtained with consent and can be viewed in Figure 1 and Figure 2.

At the time of presentation the working diagnoses included vasculitis secondary to recent NSAID usage or other collagen vascular disorders versus possible cardiac origin causing thromboemboli. She was initially started on a heparin drip to combat the possibility for further thromboemboli, and solumedrol at 1 mg/kg intravenously daily for possible vasculitic etiology. After the initiation of corticosteroids her ESR decreased from > 140 to 56 to 47 by day three. Her admission laboratory values can be viewed in Additional File 2.

Initial imaging studies included arterial ultrasound of the right lower extremity due to the color changes in her skin and pain in the extremity even at rest. Arterial doppler ultrasound demonstrated posterior tibial artery and dorsalis pedis artery with biphasic wave forms with systolic flow in normal ranges without ischemia. Radiographs of her bilateral hands and right ankle due to her complaint of joint pain showed soft tissue swelling with no air in the soft tissues or destructive lesions.

Rheumatology ordered the following laboratory studies including antinuclear antibodies (ANA), antineutrophil cytoplasmic antibodies (ANCA) with reflex MPO and PR-3, ESR, cryoglobulins, hepatitis panel, rheumatoid factor, anti-cyclic citrullinated peptide antibody (anti-CCP), serum protein electrophoresis (SPEP), dsDNA, HIV, C_3_, C_4_, uric acid and thyroid-stimulating hormone (TSH). Results of these studies were within normal limits except for SPEP which revealed a monoglonal gammopathy with elevated quantitative IgE.

Despite heparin therapy with PTT in therapeutic ranges, on the second hospital day our patient's clinical condition worsened with an increased pain and loss of palpable or doppler right pedal pulse. Her physical examination demonstrated increased non-pitting edema, loss of right light sensation, and worsening of pain. Computed tomography (CT) angiogram of her abdominal aorta with runoff to the lower extremities revealed abrupt occlusion of her right anterior tibial, posterior tibial and peroneal artery within 5 cm of their origin. There was no indication of filling defect suggestive of emboli proximal to the plane of occlusion. Given the progression of her disease over the initial days, other consideration for diagnosis included neutrophilic dermatosis, purpura fulminans with associated disseminated intravascular coagulation (DIC), and antiphospholipid syndrome or other coagulopathies. Additional labarotoy studies ordered were antithrombin III, protein C and protein S, which all showed normal results. Our patient's platelets remained stable at 200 to 380 thousand range for the entirety of her hospitalization.

Vascular surgery had an initial impression that our patient had a cardiac etiology resulting in the vascular changes in her extremities. Trans-thoracic echocardiogram (TTE), and then transesophageal echocardiogram (TEE), were negative for cardiac vegetations or other source for cardiac thromboemboli. After a cardiac source of thromboemboli was ruled out, her solumedrol medication was increased to 1 gm/kg intravenously daily for three days, and then resumed at 120 mg intravenous daily.

Culture of her urine sample grew out gram-negative rods (*Escherichia coli*). Blood cultures also grew out gram-negative rods with *E. coli *as the infectious organism. Due to bacteremia, our patient was started on intravenous antibiotics including rocephin 1 gm daily and ciprofloxacin 750 mg intravenous twice daily. Culture sensitivities returned two days later showed sensitivity to rocephin, thus prompting us to discontinue her ciprofloxacin medication.

**Figure 3:**
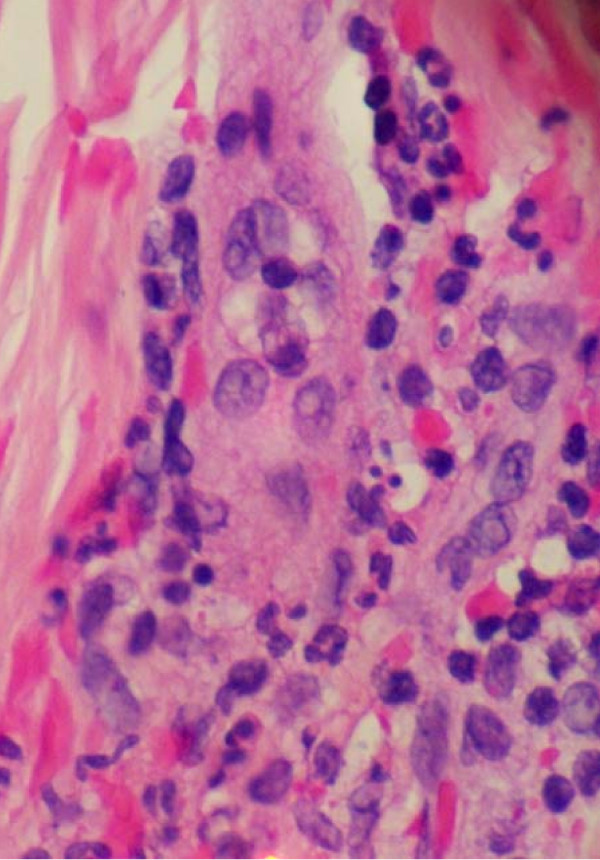
**Left thenar punch biopsy pathology high power**. Leukocytoclastic vasculitis high power view of hemotoxylin and eosin stained skin biopsy. Sections examined in multiple layers show vessels in the upper dermis in which there is destruction of the vessel wall with what appears to be fibrin and large collections of neutrophils. The picture is typical of leukocytoclastic vasculitis.

**Figure 4:**
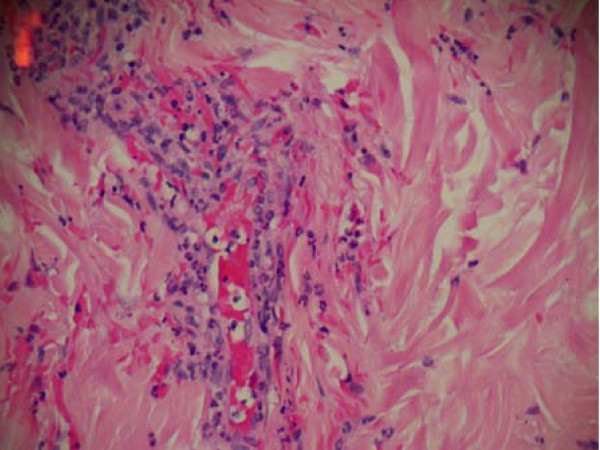
**Left thenar punch biopsy pathology low power**. Leukocytoclastic vasculitis low power view of hemotoxylin and eosin stained skin biopsy. Sections examined in multiple layers show vessels in the upper dermis in which there is destruction of the vessel wall with what appears to be fibrin and large collections of neutrophils. The picture is typical of leukocytoclastic vasculitis.

Dermatology performed a four mm-punch biopsy from her left thenar area. The results are shown in Figure 3 and Figure 4. Sections examined in multiple layers show vessels in the upper dermis in which there is destruction of the vessel wall with what appears to be fibrin and large collections of neutrophils. The picture is typical of leukocytoclastic vasculitis.

Once the diagnosis of LCV was established, we started our patient on colchicine 0.6 mg by mouth twice daily. A trial of dapsone 50 mg by mouth daily, then 100 mg by mouth twice daily was initiated on day seven, but there was a subsequent decrease in hemoglobin level by > 2 gm/dL from 11.6 to 9.3 gm/dL. As this can be an adverse effect from the dapsone, we decided to discontinue the medication. After her bacteremia was treated with intravenous antibiotics and resolved, Cytoxan (cyclophosphamide) 750 mg/m^2 ^were initiated. Our patient was also started on prophylactic bactrim to prevent *Pneumocystis jiroveci*. During the course of her treatment and trial of the above pharmaceuticals, she still did not have optimal response as her gangrene continued to progress. As a final therapeutic attempt, she was started on Revatio (sildenafil) 20 mg by mouth three times daily. During this time she was suffering peroneal nerve paralysis with loss of dorsiflexion of foot and sensory losses along with absent pulses and blackening of her toes. She did receive some benefit from multiple therapies with mild regression of involved areas to both hands and leg, but ultimately amputation was required to avert life-threatening septicemia and worsening rhabdomyolysis with peak myoglobin 2294 ng/mL (normal: 25 to 58 ng/mL), CPK 9975 U/L (normal: 26 to192 U/L), BUN 16 mg/mL (normal: 6 to 20 mg/mL), creatinine < 0.5 mg/dL (normal: 0.5 to 0.9 mg/dL) and leukocytes 25.37 K/uL (normal: 4.5 to 11.0 K/uL).

Our patient underwent amputation of her right lower extremity (below the knee amputation) on hospital day 13. Within two days her leukocytosis improved to normal range. On day 30 she underwent left second and right third finger amputation at the distal interphalangeal joints (DIPs). A final pathology of the amputated leg showed ischemic necrosis and dry gangrene. The middle and smaller blood vessels demonstrated a vasculitis similar in appearance to previous punch biopsy with neutrophilic infiltrate and fibrin deposit. There were no thrombotic or atherosclerotic occlusions, and myositis with no pyomyositis was noted. The fingers demonstrated a late stage vasculitis with thrombotic occlusion and remnants of acute inflammation with necrotic small and middle vessels.

Our patient was sent for physical rehabilitation for a short time, and then discharged home on a two-week prednisone taper.

## Discussion

Hypersensitivity vasculitis due to drugs can be identified on the basis of five defining characteristics: (1) age>16 years, (2) use of possible offending drug in temporal relation to the symptoms, (3) palpable purpura, (4) maculopapular rash, and (5) biopsy of the skin showing neutrophils around an arteriole or venule [4]. In this case there was a temporal relation between the onset of clinical deterioration of our patient and the use of a known offending agent, skin manifestations of palpable purpura, rash, biopsy of leukocytoclasis. A diagnosis of drug-induced naproxen [Naprosyn] leukocytoclastic vasculitis was thus made.

The other less likely entities, far more commonly associated with severe outcomes such as amputation, had been eliminated from the differential diagnosis based on collective consideration of the clinical, histopathologic and serologic data concerning our patient. These included the possibilities of neutrophilic dermatosis, idiopathic purpura fulminans, and necrotizing fasciitis.

Neutrophilic dermatosis, which is also a reactive hypersensitivity process that occurs in response to systemic factors such as infection, inflammation, hematologic abnormalities, vaccination or drug exposure is primarily neutrophil mediated, associated with neutrophilia and responds to medications that affect neutrophil activity [12]. Like in our case, it also can be of abrupt onset but usually consists of tender, red-to-purple papules, and nodules that coalesce to form plaques on the upper extremities, face, or neck, but not usually the legs [12]. The classic histopathologic pattern consists of a dense, diffuse neutrophilic infiltrate in the reticular dermis [12]. Leukocytoclastic nuclear debris is typically present interstitially with massive papillary dermal edema [12]. Vasculitic changes (expansion of post-capillary venule wall with fibrin deposition, as in our case) are typically absent [12]. The epidermis is also usually spared and in essentially all instances, cases with subcutaneous involvement occur with extensive involvement of the reticular dermis [12].

The diagnosis of purpura fulminans was likewise considered, and also eliminated from the differential as it is a rare syndrome of intravascular thrombosis and hemorrhagic infarction of the skin that is rapidly progressive and often accompanied by vascular collapse and disseminated intravascular coagulation [13]. It usually occurs in children, but an idiopathic form can follow an initial febrile illness and manifests with rapidly progressive purpura that leads to skin necrosis, gangrene of the limbs or digits, and major organ dysfunction [13]. It is sudden in onset, and accompanied by coagulation factor abnormalities such as undetectable levels of free protein S [13]. In over 90% of cases reported, it begins seven to 10 days after the onset of an infection with lesions beginning as erythematous macules that progress within hours to sharply defined areas of purpura [13]. Hemodynamic stability, which our patient enjoyed throughout her hospitalization, is extremely rare [13].

Lastly, necrotizing fasciitis often requires amputation and is characterized by widespread necrosis of the subcutaneous tissues and fascia [14]. It was originally believed to be a monomicrobial infection, usually with group A beta-hemolytic streptococcus and associated with some underlying cause, such as diabetes mellitus. During the last two decades, however, scientists have found that the pathogenesis of necrotizing fasciitis is polymicrobial [14,15]. The diagnosis of necrotizing fasciitis is usually made with imaging such as magnetic resonance whereby the presence of air within the tissues is detected [14,15]. Percutaneous aspiration of the soft tissue infection with gram staining of the biopsy is usually diagnostic [15]. Intravenous antibiotic therapy with early surgical fasciotomy and debridement are common [15]. The rate of death due to necrotizing fasciitis is very high, 20% to 40%, without a timely diagnosis and correct therapy [14,15]. Our patient demonstrated a worsening gangrenous state, but with the induction of therapy the areas of involvement decreased and her skin remained intact without ulceration or subcutaneous air, which is contrary to necrotizing fascitis that typically has progressively advancing borders and muscular involvement, subcutaneous air, and undermining of the epidermis [14]. Also, surgery and pathology indicated intact fascial plains with edema, and the absence of pyomyositis.

Every attempt in the treatment of vasculitis was implemented in an effort to spare our patient from amputation, which is by no accepted evidence a mainstay course of management in LCV. However, given the extenuating circumstances in this case, beginning with her progression to myoglobinemia, adverse response to some of the agents and the complication of bacteremia, the end result required amputation.

Corticosteroids are widely used in vasculitic and rheumatologic disorders [9,16]. The primary rationale is to control the local inflammatory response that is causing the ischemia [9,16]. Corticosteroids inhibit both the chemotactic response and the macrophage and neutrophilic binding to endovascular walls [9]. Corticosteroids also reduce the production of phospholipase A_2_, a key enzyme in the synthesis of arachidonic acid [9]. Arachidonic acid is the backbone for the production of prostaglandins and leukotrienes which enhance local inflammatory response [12]. Solu-Medrol (methylprednisolone) utilized at 1 gram intravenous daily is typically utilized for the initiation therapy of severe systemic vasculitis [9,16,17].

Colchicine interferes with microtubule growth of leukocytes by increasing cyclic adenosine monophosphate (cAMP), thus limiting chemotaxis and phagocytosis of neutrophils and preventing further recruitment and degranulation of lysosomes [18]. The increase in cAMP leads to the release of prostaglandin E, which further suppresses leukocyte activity [18].

Dapsone can be utilized alone or in combination with other drug modalities in the treatment of vasculitis [19]. The therapeutic property of dapsone is not derived from its antibacterial action but from a proposed inhibition and stabilization of neutrophil lysosomal enzymes [19]. It is also an antioxidant that neutralizes reactive oxygen intermediates secondary to neutrophil degranulation [19]. As such, damage from neutrophils secondary to Type III hypersensitivity can be augmented through the use of dapsone [19].

Bacteremia caused by *E. coli *secondary to a urinary tract infection delayed the initiation of chemotherapeutic pharmaceuticals such as cyclophosphamide. Concomitant use of a chemotherapeutic in patients with bacteremia would predispose them to further infection and sepsis. Cyclophosphamide remains the mainstay therapy for systemic vasculitides for the initial induction of remission [9,17]. Cyclophosphamide is a cytotoxic agent that achieves cytotoxic effects by alkylating DNA of rapidly proliferating cells [17]. In this manner it can be utilized to diminish the immune response associated with vasculitis as well as other rheumatologic disorders [9,16]. When cyclophosphamide is utilized, *Pneumocystis jiroveci *prophylaxis with trimethoprim-sulfmethoxazole should be implemented [17].

Sildenafil is a phosphodiesterase-5 inhibitor well known for use in erectile dysfunction and pulmonary hypertension. It leads to the dilation of arteries associated with digital ischemia in an attempt to restore or improve tissue perfusion during active inflammatory disorders of vessels [16].

## Conclusion

We have presented an atypical case of leukocytoclastic vasculitis in a 33-year-old African American woman secondary to the use of naproxen resulting in multi-limb ischemia and subsequent amputation. Adding yet another pharmaceutical to the list of potential causes of leukocytoclastic vasculitis will significantly add to our understanding of the etiology of this disease.

Noting the atypical nature of the case of LCV or HSV, the authors feel that the realization of paucity of cases with more severe outcomes may encourage additional research in the area of vasculitis, particularly LCV and HSV, which remains a relatively heterogeneous topic. We in no way contend that amputation should be the mainstay therapy for vasculitis, especially LCV, but based on clinical, laboratory, and multispecialty collaboration, no alternative diagnosis applied to our patient. We reiterate that amputation in this scenario was an unfortunate and debilitating last resort once all therapeutic modalities failed to improve her gangrene.

This is an original case report of particular interest to rheumatology. We were unable to find in the literature any other case of leukocytoclastic vasculitis resulting from the use of naproxen [Naprosyn].

## Consent

Written informed consent was obtained from our patient for publication of this case report and any accompanying images. A copy of the written consent is available for review by the Editor-in-Chief of this journal.

## Competing interests

The authors declare that they have no competing interests.

## Authors' contributions

KB wrote the introduction, case presentation, discussion and conclusion of the manuscript, compiled the reference list, and created the tables. JM wrote the abstract, case presentation and discussion and conducted journal and literature research. SZ reviewed the case presentation and added to the treatment of leukocytoclastic vasculitis and discussion. All authors read and approved the final manuscript.

## Supplementary Material

Additional file 1 **American College of Rheumatology criteria for hypersensitivity vasculitis**. The American College of Rheumatology criteria for the diagnosis of hypersensitivity vasculitis. Click here for file

Additional file 2 **Admission laboratory values**. The laboratory values attained on admission to the hospital. Click here for file

## References

[B1] Daniel CL Leukocytoclastic Vasculitis and Henoch-Schönlein Purpura Arthritis & Allied Conditions 2005 86 15 1793 1797

[B2] Ansari JK Hussain T Nasseem A Etodolac-induced hypersensitivity vasculitis with digital gangrene J R Coll Physicians Edinb 2007 37 205 206

[B3] Sams WM Hypersensitivity angiitis J Invest Dermatol 1989 93 785 815 10.1111/1523-1747.ep12581075 2666526

[B4] Calabrese LH Michel BA Bloch DA Arend WP Edworthy SM Fauci AS Fries JF Hunder GG Leavitt RY Lie JT The American College of Rheumatology 1990 criteria for the classification of hypersensitivity vasculitis Arthritis Rheum 1990 33 8 1108 1113 2202309 10.1002/art.1780330808

[B5] Grunwald MH Avinoach I Amichai B Halevy S Leukocytoclastic vasculitis: correlation between different histologic stages and direct immunofluorescence results Int J Dermatol 1997 37 349 352 10.1111/j.1365-4362.1997.tb03094.x 9199981

[B6] Ratnam KV Boon YH Pank BK Idiopathic hypersensitivity vasculitis: clinicopathologic correlation of 61 cases Int J Dermatol 1995 34 786 789 10.1111/j.1365-4362.1995.tb04398.x 8543412

[B7] García-Porrúa C Gonzalez-Gay MA López-Lázaro L Drug associated cutaneous vasculitis in adults in northwestern Spain J Rheumatol 1999 26 1942 1944 10493674

[B8] Martinez-Taboada VM Blanco R Garcia-Fuentes M Rodriguez-Valverde V Clinical features and outcome of 95 patients with hypersensitivity vasculitis Am J Med 1997 102 186 191 10.1016/S0002-9343(96)00405-6 9217569

[B9] Taylor HG Samanta A Treatment of Vasculitis Brit J Clin Pharmacol 1993 35 93 104 10.1111/j.1365-2125.1993.tb05674.x PMC1381499 8443041

[B10] Tosca N Stratigos JD Possible pathogenetic mechanisms in allergic cutaneous vasculitis Int J Dermatol 1998 27 5 291 296 10.1111/j.1365-4362.1988.tb02353.x 3292447

[B11] Ekenstam EA Callen JP Cutaneous leukocytoclastic vasculitis: clinical and laboratory features of 82 patients seen in private practice Arch Dermatol 1984 120 484 489 10.1001/archderm.120.4.484 6703752

[B12] Del Pozo J, Sacristán F Martínez W Paradela S Fernández-Jorge B Fonseca E Neutrophilic dermatosis of the hands: presentation of eight cases and review of the literature J Dermatol 2007 34 243 247 10.1111/j.1346-8138.2007.00261.x 17352721

[B13] Edlich RF Cross CL Dahlstrom JJ Long WB Modern concepts of the diagnosis and treatment of purpura fulminans J Env Pathol Toxicol Oncol 2008 27 3 191 196 10.1615/jenvironpatholtoxicoloncol.v27.i3.30 18652566

[B14] Costanzo M Caruso LA Condorelli F Cassaro V Longo F Forzisi G Cannizzaro MA Necrotizing fasciitis: a case report Ann Ital Chir 2008 79 4 299 302 19093634

[B15] Wang YS Wong CH Tay YK Staging of necrotizing fasciitis based on the evolving cutaneous features Int J Dermatol 2007 46 1036 1041 10.1111/j.1365-4632.2007.03201.x 17910710

[B16] Uppal SS Management of vasculitis APLAR J Rheumatol 2004 7 158 166 10.1111/j.1479-8077.2004.00079.x

[B17] Hellmann DB Imboden JB Jr Current Medical Diagnosis and Treatment 2009 48 20 New York: Lange Medical Books/McGraw-Hill 708 765

[B18] Sullivan TP King LE Boyd AS Colchicine in dermatology J Am Acad Dermato 1998 39 993 999 10.1016/S0190-9622(98)70275-0 9843014

[B19] Wolf R Tuzun B Tuzun Y Dapsone: unapproved uses or indications Clin Dermatol 2000 18 37 53 10.1016/S0738-081X(99)00093-0 10701085

