# Supplementary material for: Severe leukocytoclastic vasculitis secondary to the use of a naproxen and requiring amputation: a case report
Source: J Med Case Rep. 2010 Jul 1;4:204. doi: 10.1186/1752-1947-4-204 (PMC2906497; doi:10.1186/1752-1947-4-204)
Supplement: Additional file 1 — American College of Rheumatology criteria for hypersensitivity vasculitis. The American College of Rheumatology criteria for the diagnosis of hypersensitivity vasculitis. [file 1752-1947-4-204-S1.DOC]

**APPENDIX 1 - American College of Rheumatology Criteria for Hypersensitivity Vasculitis**

1. Age > 16 years at disease onset
2. Medication at disease onset as precipitating factor
3. Palpable purpura
4. Maculopapular rash
5. Skin biopsy with granulocytes around an arteriole or venule

For > 3 criteria: sensitivity 71% and specificity 84% for the diagnosis of HSV

Adapted from Calabrese LH, Michel BA, Bloch DA. et al. The American College of Rheumatology 1990 Criteria for the classification of Hypersensitivity Vasculitis.^3^
