# Supplementary material for: Severe leukocytoclastic vasculitis secondary to the use of a naproxen and requiring amputation: a case report
Source: J Med Case Rep. 2010 Jul 1;4:204. doi: 10.1186/1752-1947-4-204 (PMC2906497; doi:10.1186/1752-1947-4-204)
Supplement: Additional file 2 — Admission laboratory values. The laboratory values attained on admission to the hospital. [file 1752-1947-4-204-S2.DOC]

**TABLE 1 - Admission Labs**

WBC 7.79 K/uL

HGB 13.6 g/dL

HCT 37.8 %

PLT 324 K/uL

NEUT 68.7 %

EOS 0.8 %

**ESR >140 mm/hr**

GLUC 104 mg/dL

BUN 5 mg/dL

CREATININE 0.6 mg/dL

SODIUM 136 mmol/L

**POTASSIUM 3.1 mmol/L**

CHLORIDE 98 mmol/L

**CO_2_ 34 mmol/L**

TOTAL PROT 7.8 g/dL

ALBUMIN 3.8 g/dL

CALCIUM 9.8 mg/dL

T BILI 0.8 mg/dL

ALK PHOS 62 U/L

AST 17 U/L

ALT 18 U/L

MYO 21 ng/mL

TROP <0.04 ng/mL

Abnormal laboratory values are noted in bold text.
